# The Induction of Noble Rot (*Botrytis cinerea*) Infection during Postharvest Withering Changes the Metabolome of Grapevine Berries (*Vitis vinifera* L., cv. Garganega)

**DOI:** 10.3389/fpls.2017.01002

**Published:** 2017-06-21

**Authors:** Stefano Negri, Arianna Lovato, Filippo Boscaini, Elisa Salvetti, Sandra Torriani, Mauro Commisso, Roberta Danzi, Maurizio Ugliano, Annalisa Polverari, Giovanni B. Tornielli, Flavia Guzzo

**Affiliations:** ^1^Biotechnology Department, University of VeronaVerona, Italy; ^2^Unione Italiana Vini Soc. coopVerona, Italy

**Keywords:** postharvest withering, Garganega grapes, noble rot induction, metabolomics, VOCs

## Abstract

The natural or induced development of noble rot caused by the fungus *Botrytis cinerea* during the late stages of grapevine (*Vitis vinifera* L.) berry ripening is used in some traditional viticulture areas to produce high-quality wines such as Sauternes and Tokaji. In this research, we wanted to verify if by changing the environmental conditions during post-harvest withering we could induce the noble rot development on harvested berries in order to positively change the wine produced from withered Garganega berries. Therefore, we exposed the berries to postharvest withering under normal or artificially humid conditions, the latter to induce noble rot. The presence of noble rot symptoms was associated with the development of *B. cinerea* in the berries maintained under humid conditions. The composition of infected and non-infected berries was investigated by untargeted metabolomics using liquid chromatography/mass spectrometry. We also explored the effects of the two withering methods on the abundance of volatile organic compounds in wine by yeast-inoculated micro-fermentation followed by targeted gas chromatography/mass spectrometry. These experiments revealed significant metabolic differences between berries withered under normal and humid conditions, indicating that noble rot affects berry metabolism and composition. As well as well-known botrytization markers, we detected two novel lipids that have not been observed before in berries infected with noble rot. Unraveling the specific metabolic profile of berries infected with noble rot may help to determine the compounds responsible for the organoleptic quality traits of botrytized Garganega wines.

## Introduction

The necrotrophic ascomycete *Botrytis cinerea* has been described as a ‘Jekyll and Hyde’ fungus because it causes devastating gray mold disease in grapevine plants but is also responsible for noble rot in ripe and overripe berries, which allows the production of high-quality sweet wines such as Sauternes and Tokaji (Fournier et al., 2013). Gray mold caused by *B. cinerea* is one of the most severe grapevine diseases, reducing both the quality and quantity of berries. The resulting wines are poor because the infected berries have an unfavorable composition and the pathogen also produces toxic compounds that affect yeast and thus inhibit the fermentation process (Bocquet et al., 1995; Hong et al., 2011; Agudelo-Romero et al., 2015). However, the development of *B. cinerea* as noble rot (botrytization) is a favorable process lasting 10–20 days and is typical of particular wine productions. The berry is transformed by the penetration of fungi through stomata, wounds or microfissures on the fruit surface (the *pourri plein* stage), the permeabilization of the fruit skin encouraging water loss and sugar concentration, and finally enzymatic maceration (the *pourri rôti* stage) (Ribéreau-Gayon et al., 2006). At the end of this process, further fungal development is arrested by the high sugar concentration and, if still on the plants, the botrytized berries can be harvested individually. Noble rot confers a berry composition which is distinct from that of berries with gray rot and uninfected berries and is potentially associated with desirable aroma characters of the resulting wine. In addition to directly producing potent odorants such as phenylacetaldehyde, lactones and vanillin (Lopez Pinar et al., 2016), noble rot infection can indeed stimulate production, in the berry, of cysteine and glutathione conjugates which can be transformed by the yeast into the powerful aroma compound 3-mercaptohexanol (Thibon et al., 2009, 2011). The developmental transition between gray rot and noble rot is influenced by environmental conditions and soil characteristics. Moist nights, foggy mornings and dry, sunny days promote the slow infection that results in noble rot, whereas strong rainfall and high humidity facilitate the more aggressive gray mold (Ribéreau-Gayon et al., 1980; Gubler et al., 2013).

Recioto di Soave is an Italian *passito* wine (i.e., a wine produced from dehydrated grapes) made from the white-skinned berries of the cultivar Garganega. Grape dehydration (known as withering) takes place after harvest in a dedicated room known as the *fruttaio*. Slow postharvest withering can favor noble rot development, induced by particular environmental conditions and/or artificial *B. cinerea* inoculation, thus allowing botrytization to be implemented in regions where natural noble rot is uncommon (Lorenzini et al., 2012; Tosi et al., 2013).

The metabolomic and transcriptomic changes that occur in black-skinned grape berries of various cultivars during traditional postharvest withering in *fruttaio* have recently been described (Zenoni et al., 2016). The behavior of withering berries is strongly cultivar dependent. The berries of slow-withering cultivars are more metabolically active during the process, showing both *de novo* synthesis of various metabolites (especially stilbenes) and a higher number of differentially expressed genes. Transcriptomics and metabolomics have also been used to investigate the response of white-skinned Sémillon berries during noble rot infections (Blanco-Ulate et al., 2015). The fruits of this cultivar respond to *B. cinerea* infection by upregulating genes involved in the response to pathogens and stress, fruit ripening, and hormone metabolism, and by accumulating certain secondary metabolites such as phenylpropanoids and terpenes.

Here we used untargeted metabolomics based on liquid chromatography/mass spectrometry (LC-MS) to characterize the metabolites of Garganega berries during postharvest withering *in fruttaio*, under standard conditions and with artificial humidification used to induce *B. cinerea* colonization. We also used targeted gas chromatography/mass spectrometry (GC-MS) to characterize the volatile organic compounds (VOCs) in wines produced from berries exposed to the two postharvest withering treatments.

## Materials and methods

### Withering methods and berry sampling

Approximately 170 kg of Garganega berries was harvested at the commercial ripening stage (soluble solids content = 18.5 ± 0.25°Brix) in Monteforte d'Alpone (Verona, Italy) at the beginning of October and transported to the Pasqua Vigneti e Cantine winery. The berries were placed in perforated plastic boxes (*plateaux*, ~5 kg in each) in a ventilated withering facility under natural conditions (17–20°C, 78–82% relative humidity) and sampled (T0).

Brix degrees were measured weekly in three randomly selected replicates using a DBR35 digital refractometer (Giorgio Bormac, Carpi, Italy). Three boxes were also weighed weekly using a CH50K50 electronic balance (Kern, Balingen, Germany) in order to determine the weight loss of berry bunches during withering. After 29 days, when the weight loss was ~30% of the initial weight, grape clusters were sampled (T1) and half of the *plateaux* were covered with plastic film at 15–17°C. Water-filled trays were placed inside to increase the relative humidity (88–94%) and encourage *B. cinerea* development. The remaining *plateaux* were left under normal withering conditions (15–17°C, 68–75% relative humidity). The two different environmental conditions were imposed for 32 days before the final samples were taken obtaining T2-n (normal withering, ventilated) and T2-i (induced noble rot) samples. The total duration of dehydration was 61 days. The relative humidity and temperature inside and outside the covering were monitored using Hobo Pro v2 sensors connected to data loggers (Onset Computer Corporation, Bourne, MA, USA).

**Figure 1:**
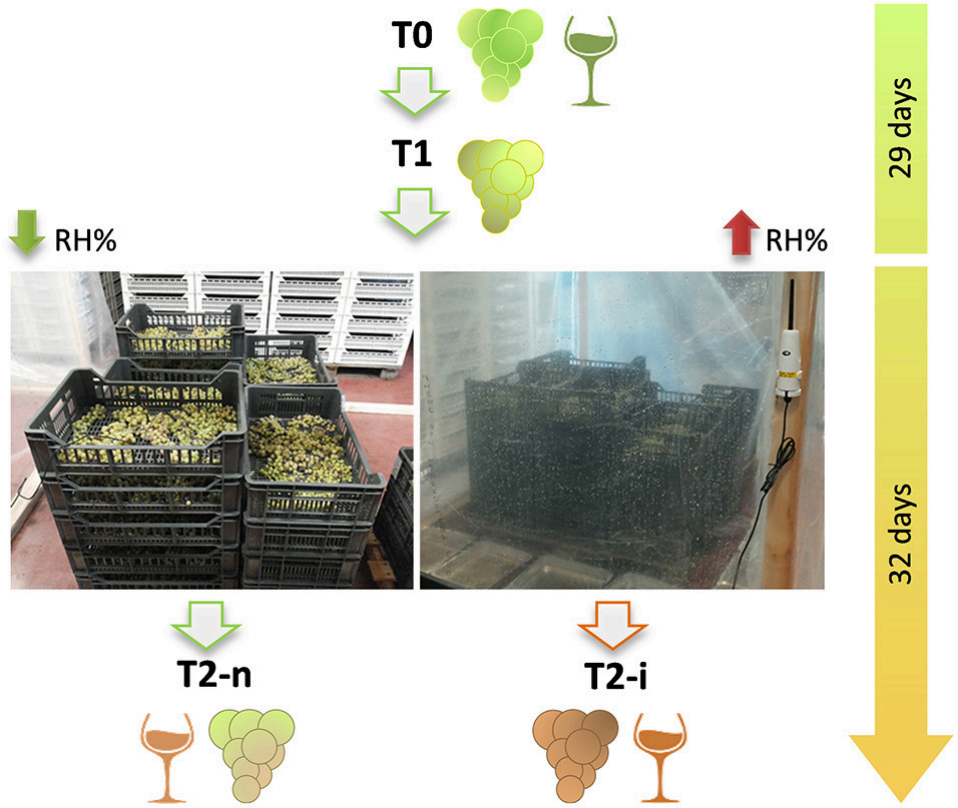
The two types of withering process applied to Garganega berries. Harvested berries were withered under natural conditions for 29 days and then half of the berries were enclosed in plastic crates (right-hand image) to increase the relative humidity (RH%) and favor *Botrytis cinerea* development in the form of noble rot. The whole withering process lasted 61 days. T0 = berries at the beginning of withering; T1 = withering berries collected before varying the humidity; T2-n = naturally withered berries; T2-i = berries withered under higher humidity conditions to induce noble rot.

Three independent pools of 3 kg of berries each were collected for each treatment and used in the following analysis. For T0, T1 and T2-n grape berries were randomly sampled whereas for T2-i berries were visually selected for noble rot symptoms. Each sample was used to determine the average berry weight, the number of *B. cinerea* colony forming units (CFUs) and for LC-MS analysis. The remaining of T0, T2-n, and T2-i berry samples (about 2 Kg for each biological replicates) were also pressed and the resulting musts were micro-fermented followed by GC-MS analysis of the wine. A simplified experimental workflow is reported in Figure 1.

### Enumeration of *B. cinerea* CFUs

We randomly selected 100 g of berries from each biological replicate and crushed them independently. The juices were serially diluted in 25% Ringer's solution (Oxoid, Basingstoke, UK) and 100-μl aliquots were spread on triplicate plates of Botrytis Selective Medium (Edwards and Seddon, 2001). The *B. cinerea* CFUs were counted on the plates after 5–7 days of incubation at 20°C in the dark.

### Extraction of metabolites and LC-MS analysis

250 g of frozen berries for each pool was ground in liquid nitrogen and 300 mg of berry powder was extracted in three volumes of cold LC-MS-grade methanol. After mixing, the samples were sonicated for 15 min at 4°C and centrifuged (16 000 rcf, 10 min, 4°C). The supernatants were diluted 1:2 in LC-MS-grade water, passed through 0.2 μm Minisart RC4 filters (Sartorius-Stedim Biotech, Göttingen, Germany) and analyzed by reversed-phase high-performance liquid chromatography (RP-HPLC) using a Gold 127 HPLC System (Beckman Coulter, Brea, CA, USA) equipped with a C18 guard column (7.5 × 2.1 mm, 5 μm particle size) and an Alltech (Nicholasville, KT, USA) RP C18 column (150 × 2.1 mm, 3 μm particle size). A gradient between solvent A (0.5% formic acid and 5% acetonitrile in water) and solvent B (100% acetonitrile) was set as follows: 0–10% B in 2 min, 10–20% B in 10 min, 20–25% B in 2 min, 25–70% B in 7 min, isocratic for 5 min, 70–90% B in 1 min, isocratic for 14 min, 90–0% B in 1 min, and 20 min equilibration. For each sample, 20 μl was injected at a flow rate of 0.2 ml min^−1^.

The HPLC instrument was coupled on-line to an Esquire 6000 ion trap mass spectrometer equipped with an electrospray ionization (ESI) source (Bruker Daltonik, Bremen, Germany). Mass spectra were recorded in alternating positive and negative ionization mode within the range 50–1,500 *m/z* with a target mass of 400 *m/z*. Nitrogen was used as the nebulizing gas (50 psi, 350°C) and drying gas (10 L min^−1^) and the vacuum pressure was 1.4 × 10^−8^ bar. For fragmentation analysis, MS/MS and MS^3^ spectra were recorded in positive and negative ionization modes in the range 50–1,500 *m/z*. Helium was used for collision induced dissociation (amplitude = 1 V). MS data were collected using Esquire Control v5.2 software and processed using Esquire Data Analysis v3.2 software (both provided by Bruker Daltonik).

Metabolites were identified by comparing retention times, *m/z* values and fragmentation patterns with those of commercial standards in our in-house library. When no authentic standard compounds were available, identification relied on the fragmentation patterns in online databases such as MassBank (www.massbank.jp) or reported in the literature. Neutral losses of 132, 146, and 162 Da were considered diagnostic of the loss of pentose, deoxyhexose, and hexose sugars, respectively.

### Lipid extraction and LC-MS analysis

*Botrytis cinerea* strain B05.10 (Amselem et al., 2011) was inoculated into flasks containing 125 ml potato dextrose broth (Formedium, Hunstanton, UK) at a concentration of 7 × 10^6^ conidia/ml, and incubated for 7 days at 22°C, shaking at 120 rpm. *B. cinerea* mycelia were recovered by filtration and ground in liquid nitrogen. We resuspended 300 mg of frozen berry powder or *B. cinerea* mycelia in 300 μl LC-MS-grade water and then mixed the suspension with 3 ml glacial chloroform/methanol (2:1). The samples were vortexed for 30 s, stored on ice for 1 h, sonicated for 15 min and centrifuged (25 min, 4,500 rcf, 4°C). The chloroform phases (~2 ml) were recovered, placed in 2-ml plastic tubes and centrifuged again (10 min, 16,000 rcf, 4°C). The supernatants were recovered, partially evaporated in a Heto Holton Maxi-Dry Plus Vacuum (Thermo Fisher Scientific, Waltham, MA, USA) and tubes containing the same extract were pooled. Finally, the solvent was completely evaporated, the residue was resuspended in three volumes (w/v) of LC-MS-grade methanol and sonicated for 3 min. One sample, arbitrary selected as Quality Control 1 (QC1), and a methanolic solution including 1 μg/μl palmitic acid as QC2 (Sigma-Aldrich, St Louis, MO, USA) were analyzed at the beginning and end of the experiment, respectively. Finally, the solutions were passed through a Minisart RC4 0.2-μm filter and 20 μl was injected into the abovementioned LC-MS system. The solvents were 0.5% (v/v) formic acid in LC-MS-grade water (A) and 100% acetonitrile (B). A gradient was established from 50 to 100% B in 10 min, followed by 65 min under isocratic conditions and then from 100 to 50% B in 1 min. The column was finally equilibrated for 15 min. MS analysis was carried out by equipping the mass spectrometer with an atmospheric pressure chemical ionization (APCI) source and using the same parameters described above for medium-polar metabolites.

### Micro-vinification

Fermentation trials were carried out using musts from T0, T2-n, and T2-i berry samples. The musts were separated from the pomace and mixed with 0.3 g/l activating agent (Apapiù Mix, Tebaldi, Colognola ai Colli, Italy) and 15 mg/l sodium metabisulfite (Sigma-Aldrich) before transferring 500 ml of each must carefully into sterile bottles. After measuring the Brix degrees, the musts were inoculated with 10^6^ CFU/ml of the commercial wine strain *Saccharomyces cerevisiae* Mycoferm CRU 69, previously activated by following the manufacturer's instructions (Ever, Pramaggiore, Italy). Each fermentation experiment was performed in triplicate at a controlled temperature of 18°C. We monitored the fermentation kinetics for 14 days by gravimetric analysis to determine the loss of weight due to the production of CO_2_.

### GC-MS analysis of micro-vinificated wines

Volatiles were analyzed by gas chromatography–mass spectrometry (GC-MS) after solid–phase extraction (SPE). SPE was performed using ENV^+^ cartridge (1 g, 40–140 μm; Isolute, IST Ltd., Mid Glamorgan, UK) and an Aspec XL Sample Processor for SPE (Gilson Inc. Middleton, WI, USA). The cartridges were sequentially conditioned with methanol (9.5 ml) and distilled water (19 ml). A total of 38 ml of wine sample diluted 1:2 with distilled water, and 1–heptanol added as internal standard (500 μg/l) was loaded onto the cartridge. The residue was washed with 19 ml of distilled water. The free aroma compounds were eluted with 9 ml of dichloromethane. The solution was dried with Na_2_SO_4_ and concentrated to 0.4 ml by nitrogen flow stream. GC–MS analysis was performed with 6980N Network GC System coupled with a 5975 XL EI/CI MSD (Agilent Technologies, Santa Clara, CA, USA), equipped with DB–WAX Bonded PEG fused silica capillary column (60 m × 320 μm i.d. × 0.25 μm film thickness; Agilent Technologies). Instrumental conditions were: electron impact (EI) mode 70 eV; injector temperature 200°C; He carrier flow 1.5 ml/min; column temperature 50°C for 4 min, rising to 240°C at 4°C/min, then 20 min at 240°C; and injection volume 2.0 μL in splitless mode. The analyses were performed in SCAN mode. NIST data bank and co-injection of pure reference standards were used to identify the compounds.

### Statistical analysis of samples

Statistical significance between samples analyzed for percentage of weight loss, soluble solid content, average berry weight, *B. cinerea* enumeration and fermentation kinetics was evaluated by t-student. For LC-MS and GC-MS data, raw chromatograms were converted to netCDF files for peak alignment and area extraction using MZmine software (http://mzmine.sourceforge.net/) and multivariate statistical analysis was applied to the resulting dataset using SIMCA v.13.0 (UmetrixAB, Umeå, Sweden). Pareto scaling was applied to all analytical methods. Unsupervised principal component analysis (PCA) was used to identify the major clusters defined by the samples, and two supervised methods, namely partial least squares discriminant analysis (PLS-DA) and orthogonal projections to latent structures discriminant analysis (OPLS-DA/O2PLS-DA), were used to compare classes in order to identify metabolites that characterize different withering stages. PLS-DA models were validated by a permutation test (200 permutations) and the corresponding OPLS-DA/O2PLS-DA models were cross-validated by analysis of variance (ANOVA) with a threshold of *p* < 0.01.

## Results

### Artificial humidification of berries increases the prevalence of *B. cinerea* colonization

**Figure 2:**
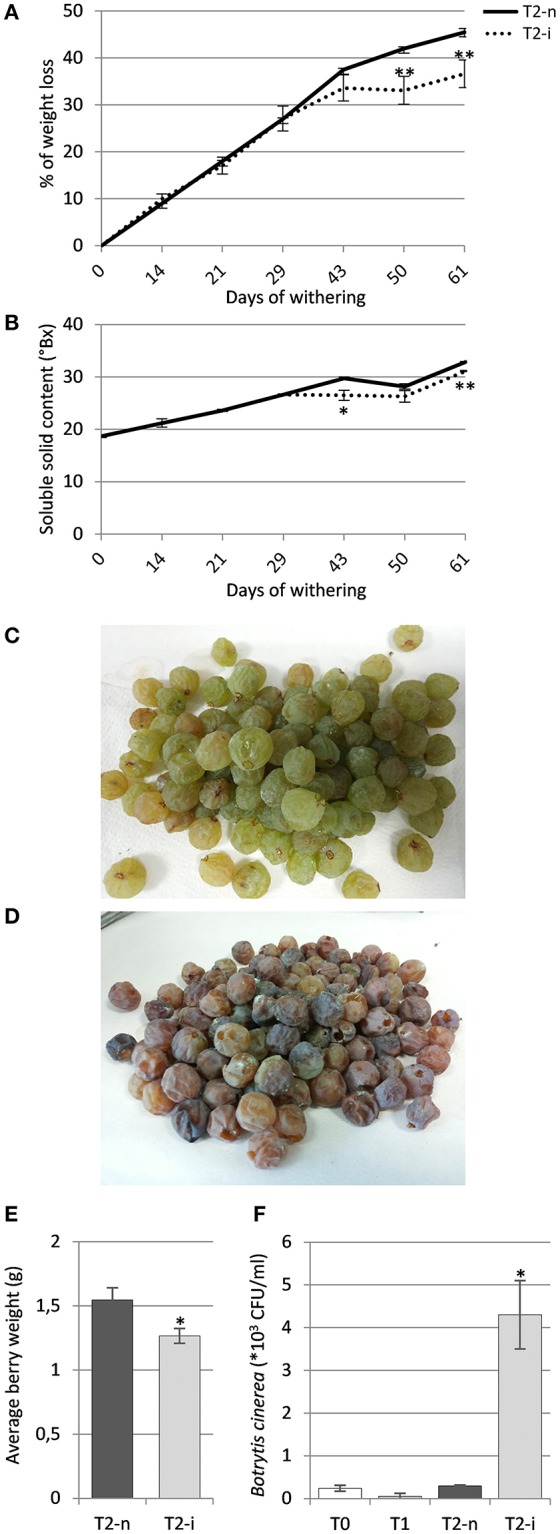
Garganega berry characteristics. **(A)** Percentage weight loss and **(B)** soluble solids (°Bx) during normal withering (T2-n) and induced noble rot (T2-i). **(C)** Appearance of sampled berries withered under natural conditions or **(D)** under higher humidity to induce noble rot. **(E)** Average berry weight in the T2-n and T2-i samples at the end of the withering process. **(F)** Enumeration of *Botrytis cinerea* colony forming units (CFUs) in samples T0, T1, T2-n, and T2-i. Vertical bars represented standard deviations (SD) of means (*n* = 3). Asterisks refer to *t*-student *p*-values obtained from T2-n and T2-i comparison (^*^p < 0.05, ^**^p < 0.01).

The berries maintained under standard conditions showed a linear weight loss during the first 40 days of withering followed by slower weight loss toward the end of the process, whereas the berries maintained under artificially high humidity lost weight more slowly (*p* < 0.01, Figure 2A). The Brix degrees increased during withering due to the loss of water and consequently the concentration of sugars, hence the profile was complementary to the loss of weight, increasing more slowly in the covered berries (Figure 2B).

At the end of the withering process, only the covered berries showed the typical symptoms of noble rot, with roughly 70% of the berries visually appearing as chocolate-brown colored, more shriveled and dehydrated compared to control berries (Figures 2C,D). These berries were visually selected for further analysis. The comparison between the average berry weight of visually selected T2-n and T2-i berries revealed that T2-i weight was slightly lower than the control berries (*p* < 0.05, Figure 2E). This was also associated with a higher prevalence of *B. cinerea* colonization as determined by counting the number of CFUs on selective medium, confirming that the conditions used for induction supported *B. cinerea* growth in its latent form (*p* < 0.05, Figure 2F). No gray mold symptoms were observed among the covered berries.

### Untargeted metabolomics reveals fungal metabolites, plant phytoalexin accumulation, and plant metabolite depletion

**Figure 3:**
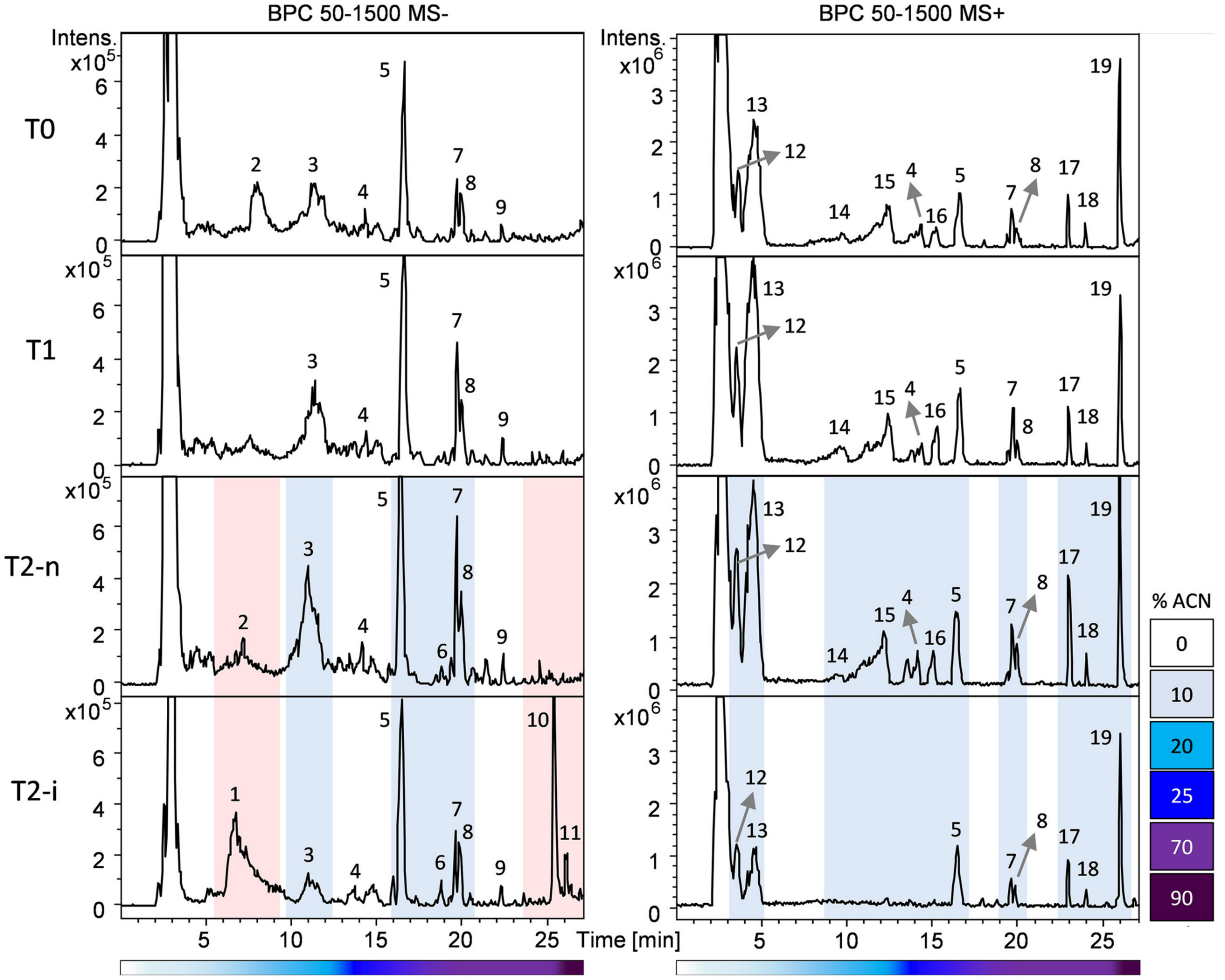
Base peak chromatograms (BPC) recorded in negative (left) and positive (right) ionization mode based on the RP-HPLC-ESI-MS analysis of T0, T1, T2-n, and T2-i berry samples. Peak numbers refer to the metabolites listed in Table 1. Chromatogram areas are highlighted to show the reduction (in blue) or the accumulation (in red) of metabolites in T2-i grapes relative to naturally withered grapes (T0, T1, T2-n). The lower bars refer to the percentage of acetonitrile (ACN) in the mobile phase.

Berries were sampled for metabolomic comparison at the beginning of the experiment (T0), 29 days later just before the two different withering conditions were applied (T1), and at the end of the experiment, separately for the berries exposed to conventional withering (T2-n) and the high humidity conditions (T2-i). Representative LC-MS chromatograms obtained in negative and positive ionization modes are shown in Figure 3. The blue zones highlight metabolites that increased in abundance during dehydration but were largely consumed in T2-i berries in comparison with T2-n berries. These metabolites included caffeoyl tartaric acid (caftaric acid), the amino acids leucine/isoleucine, phenylalanine, and tryptophan (together with its caffeic acid derivative), flavan-3-ols and flavonols. The red zones indicate metabolites that became more abundant or appeared *de novo* in the artificially humidified berries (T2-i) in comparison with T2-n berries. These included uridine 5'-diphospho-N-acetylglucosamine, a resveratrol tetramer and 13-keto-9Z,11E-octadecadienoic acid/13-oxo-9Z,11E-octadecadienoic acid (13-KODE). The metabolites present in all samples are summarized in Table 1.

**Table 1:** Main metabolites detected by RP-HPLC-ESI-MS analysis in negative ([M-H]^−^) and positive ([M+H]^+^) ionization modes.

			**[M-H]**^**−**^		**[M**+**H]**^**+**^		
**Peak n°**	**Rt**	**Putative identification**	**m/z**	**MS^2^ (MS^3^)**	**m/z**	**MS^2^ (MS^3^)**	**References**
1	6.7	Uridine 5′-diP-N-acetylglucosamine^**^	605.9	384.8 (nf), 402.7, 281.8, 272.7	−	−	MassBank
2	7.9	2-S-Glutathionylcaftaric acid	616.0	439.9 (271.7, 166.8, 142.8), 253.8	−	−	Boselli et al., 2006
3	10.0	Caffeoyl tartaric acid^*^	310.9	148.7 (86.9, 130.7, 102.8), 178.7	−	−	Library
4	14.3	Catechin derivative^**^	435.0	136.8 (108.7), 288.8, 270.8, 244.8	437.1	285.1 (163.0, 249,1, 205.0), 267.1	Putative identification
5	16.5	Caffeic acid tryptophan^**^	366.1	185.8 (141.8), 203.8, 245.7, 217.8	385.1	−	Putative identification
6	18.8	Resveratrol hexose isomer I	389.1	226.8 (184.7, 158.8), 227.7, 164.8	−	−	Sandhu and Gu, 2010
7	19.6	Quercetin-3-O-glucoside^**^	463.0	300.7 (150.8, 299.8, 178.7), 301.7	465.0	303.0 (257.0, 229.0, 153.0, 165.0)	Library
8	19.8	Quercetin-glucuronide^*^	477.0	300.8 (178.6, 150.6, 151.7), 301.7	479.0	303.0 (229.0, 214.7), 317.0, 304.1	Hvattum, 2002
9	22.3	Resveratrol hexose isomer II	389.0	226.8 (nf), 227.7, 228.7, 184.5	−	−	Sandhu and Gu, 2010
10	25.4	Resveratrol tetramer^*^	905.3	811.0 (717.0), 812.0, 718.0, 356.9	−	−	Püssa et al., 2006
11	26.8	13-KODE^**^	293.0	202.8 (174.8), 220.8, 148.8	−	−	MassBank
12	3.5	L-(Iso)leucine^**^	−	−	132.0	86.3 (nf), 87.3, 84.1	MassBank
13	4.6	L-Phenylalanine^**^	−	−	166.0	120.2 (79.2, 103.1), 149.1, 121.1	MassBank
14	9.7	L-Tryptophan^**^	−	−	205.0	188.0 (146.1, 144.0)	Massbank
15	12.4	(+)-Catechin^**^	−	−	291.1	139.1 (111.0), 123.1, 165.1, 147.1	Library
16	15.2	(−)-Epicatechin^**^	−	−	291.1	139.1 (111.0), 123.1, 165.1, 147.1	Library
17	22.9	Unidentified^**^	−	−	404.2	242.1 (224.1, 223.0, 96.1, 222.0)	−
18	24.0	Unidentified^**^	−	−	404.2	242.1 (nf)	−
19	26.0	Unidentified^*^	−	−	226.1	178.0 (150.1), 207.9, 181.3, 116.2	−

*Peak numbers refer to the chromatogram profiles in Figure 3. Rt, retention time (min); Nf, not fragmented. Asterisks refer to those metabolites which average peak areas was significantly different between T2-n and T2-i as assessed by t-student test*.

* *p < 0.05*,

** *p < 0.01*.

The chromatograms were used to build two data matrices (negative and positive ionization mode, respectively). The negative ionization data matrix contained 257 *m/z* features, 74 of which were tentatively identified. They included 52 metabolites plus adducts and fragments (Supplementary File 1, Datasheet 1). The positive ionization data matrix contained 356 *m/z* features, 33 of which were identified, corresponding to 22 different metabolites (Supplementary File 1, Datasheet 2).

**Figure 4:**
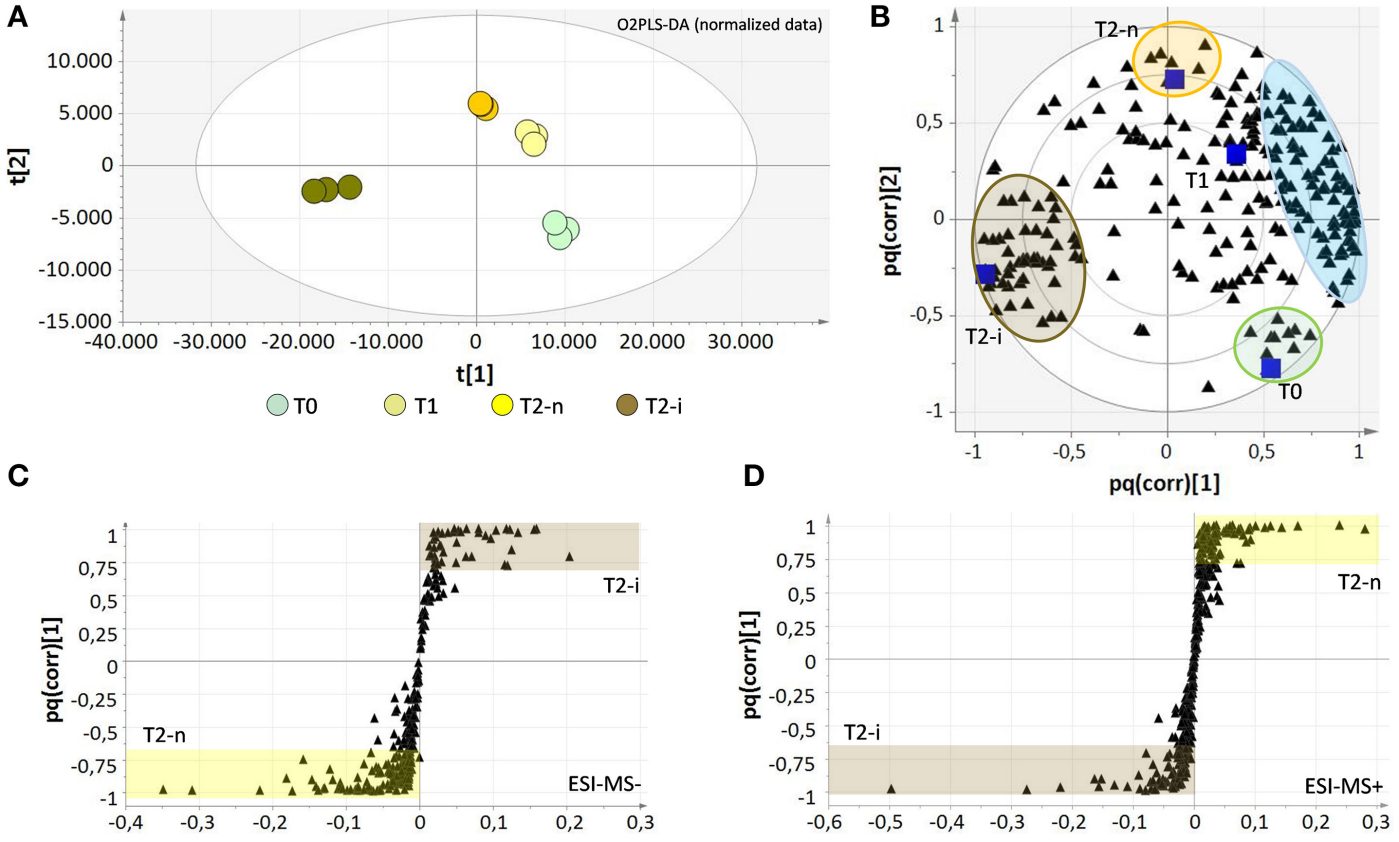
Score plots **(A)** and correlation loading plots **(B)** for the O2PLS-DA model of the negative data matrices after data normalization for sample weight loss. The metabolites which strongly characterize each sample are highlighted with colored circles and are listed in Supplementary File 2 (Datasheet 1). The light blue circle comprises all metabolites that characterize the natural withering process and negatively correlate with berries infected with noble rot (T2-i). Correlation loading plots for the OPLS-DA models of negative **(C)** and positive **(D)** data matrices show the distribution of metabolites between T2-n and T2-i berries. All metabolites with pq(corr) values > 0.7 or < −0.7 are considered highly characteristic of T2-n (highlighted in yellow) or T2-i (highlighted in brown) berries and are listed in the Supplementary File 2 (Datasheet 2).

The two data matrices were explored by multivariate analysis (O2PLS-DA). The results obtained for the negative ionization data matrix are shown in Supplementary Figures 1A,B. The O2PLS-DA loading plot was expressed as a pq(corr) value, representing the correlation between the p part of the model (the class of samples) and the q part of the model (the metabolites). The spatial closeness among the metabolites (black triangles) and the samples (blue squares) reflects their relationships, revealing the concentration effect from T0 to T1 and T2, but also specific effects in the botrytized samples that differ substantially from the non-botrytized controls (Supplementary Figure 1B). To overcome concentration effects and focus on concentration-independent effects, the data matrices were normalized for weight loss, and the signal intensities were expressed relative to the weight at the beginning of the experiment (Supplementary File 1, Datasheets 3, 4). The results following data normalization are shown for the negative data matrix (Figures 4A,B). The enrichment of metabolites previously observed in the T2-n samples is now effectively shared among the T0, T1, and T2-n samples (light blue circle) confirming these metabolites are characteristic of the entire traditional withering process. Despite this normalization effect, a few metabolites were typical of the T0 and T2-n control samples (green and orange circles, respectively). Interestingly, the botrytized berries (brown circles) were strongly characterized by a group of specific metabolites which correlate negatively with the traditional withering process. The metabolites are listed in Supplementary File 2, Datasheet 1.

OPLS-DA analysis was also applied to both the negative and positive ionization mode data matrices for the normal withering (T2-n) and botrytized (T2-i) berry samples (Figures 4C,D) better highlighting differences between T2-n and T2-i. The set of strongly characteristic metabolites identified by this analysis (Supplementary File 2, Datasheet 2) reflects the depletion and *de novo* production of metabolites in T2-i as already highlighted in Figure 3 and reported in Table 1 (e.g., resveratrol derivatives, 13-KODE and uridine 5′-diphospho-N-acetylglucosamine).

The depleted compounds represent diverse metabolite classes (e.g., sugars, amino acids, flavonoids and some stilbenes). Other metabolites accumulate rapidly in the botrytized berries, including pantothenic acid, some stilbenes (dimers, trimers and tetramers but not the monomers), glucose-6-phosphate, uridine 5′-diphospho-N-acetylglucosamine, a lipid putatively annotated as 13-KODE, and other unidentified metabolites. The presence of the N-acetylglucosamine donor uridine 5′-diphospho-N-acetylglucosamine indicates an active fungal metabolism because this sugar is a precursor of the chitin found in the fungal cell wall. Some of the molecules accumulating in botrytized berries were also clearly detectable in the chromatograms as major signals (Figure 3), including one resveratrol tetramer (Figure 3) and two metabolites with retention times of 30 and 32 min respectively (not shown). These two metabolites showed similar behavior, suggesting similar chemical properties. Both showed the chloride and formic adducts as main signals in negative ionization mode, and the molecular ions in positive ionization mode, and both generated fragments at *m/z* 355, 337, and 206. Although we were unable to identify these molecules, the higher retention time suggested they are lipids. We therefore re-extracted the methanol extracts with chloroform, and the resulting lipid fractions were analyzed by LC-APCI-MS using a method optimized for lipid analysis. The lipid profiles of T2-n and botrytized (T2-i) samples are shown in Supplementary Figure 2. This analysis confirmed the lipid-like nature of the two unidentified T2-i metabolites (highlighted in the figure) and showed that the general lipid profile is otherwise similar between the T2-n and T2-i samples. The same peaks could not be detected using the same LC-APCI-MS approach following the extraction of lipids from *B. cinerea* strain B05.10 grown *in vitro*, indicating that the two unidentified lipids might not be typical constituents of the fungus (data not shown). On the other side, we cannot exclude that the wild type *B. cinerea* strains developed in this experiments have different composition than the used reference B05.10 strain (Amselem et al., 2011).

### The wines produced by botrytized and conventionally withered berries show different aromatic profiles

**Figure 5:**
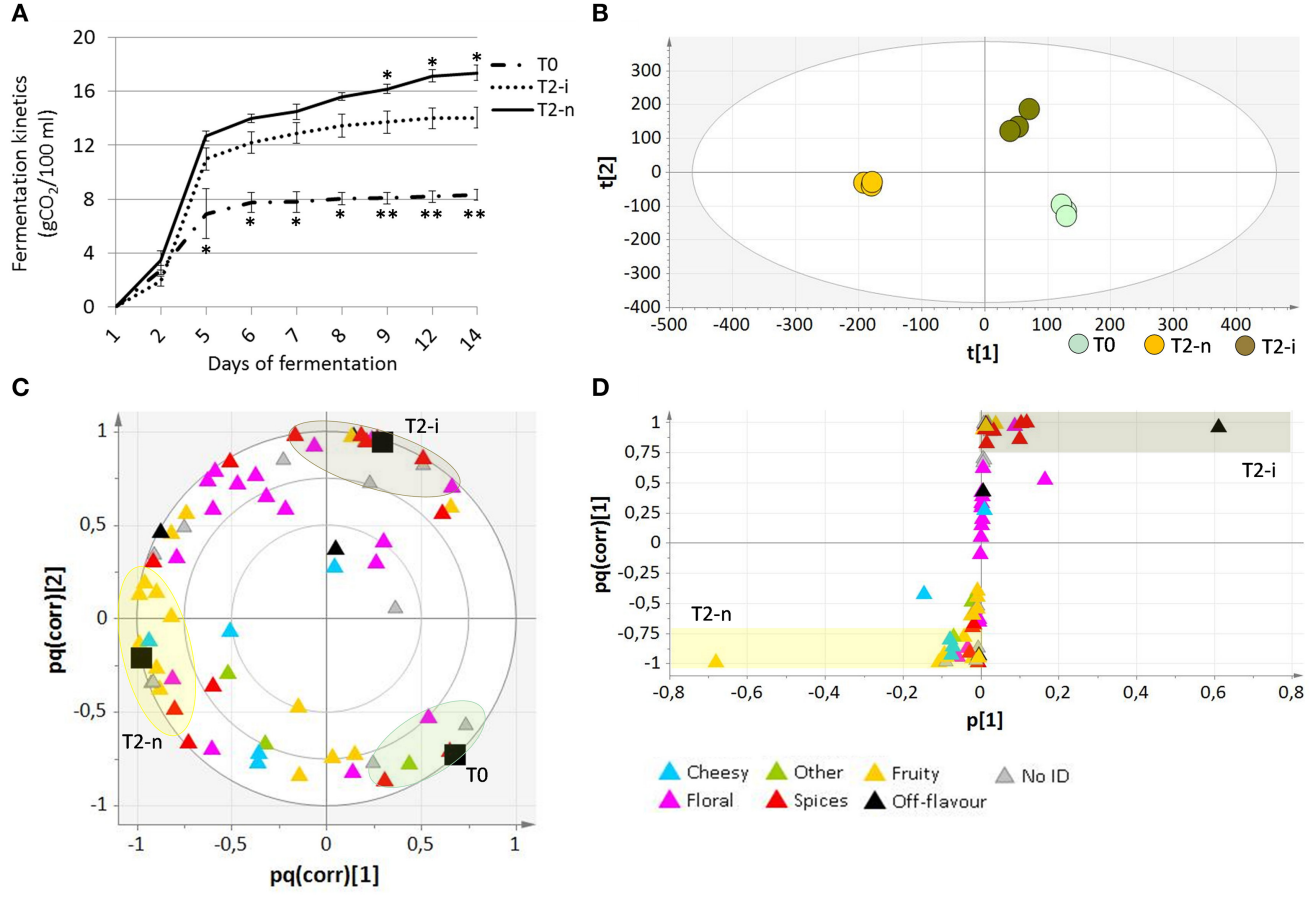
Analysis of wines produced from Garganega berries. **(A)** Fermentation kinetics of the musts obtained from T0, T2-n, and T2-i berries during the 14 days of micro-vinification. Vertical bars represented standard deviations (SD) of means (*n* = 3). Asterisks refer to *t*-student *p*-values obtained from comparison of T2-i with T-n and T0 samples (^*^p < 0.05, ^**^p < 0.01) **(B)** O2PLS-DA score plot and **(C)** loading plot of T0, T2-n, and T2-i wines. In **(C)** correlation loading of the aroma differentiation characterizing each sample is represented by a color code, with VOCs grouped according to their aromatic class. **(D)** OPLS-DA loading correlation plot of withered control (T2-n) and botrytized (T2-i) wines.

Micro-scale vinification was performed on fresh berries (T0), withered botrytized berries (T2-i) and conventionally withered berries (T2-n). The °Brix values of the musts from the T0, T2-i and T2-n berries were 18.67 ± 0.25, 31.13 ± 0.11, and 32.87 ± 0.06, respectively. The must fermentation rate (calculated as grams of CO_2_/100 ml of must) was generally higher in the musts from withered grapes (Figure 5A). Moreover, musts from the conventionally withered grapes showed a more vigorous fermentation compared to the musts from botrytized berries (*p* < 0.05).

The aromatic profile of the three wines was analyzed by GC-MS 14 days after the beginning of the vinification. The O2PLS-DA model of the entire GC-MS data matrix revealed that the three wines showed distinct aromatic compositions (Figures 5B,C). The compounds characterizing the three wines are listed in Table 2, and their pq(corr) values are shown in Supplementary File 3. Wines from T2-n grapes were mainly characterized by fruity aromas, whereas botrytized wines were characterized by spicy aromas (Figure 5C).

**Table 2:** Aroma compounds highlighted in Figure 5C characterizing T0, T2-n, and T2-i musts.

		**Aromatic features**			**ppb**		
	**VOCs**	**Molecular class**	**Descriptor**	**Class**	**T0**	**T2-n**	**T2-i**
**T0 characterizing**	Ethyl 2-hydroxy-4-methylpentanoate	Esters			29.6 ± 5.4	13.4 ± 2.6	16.3 ± 0.7
	Ethyl lactate	Esters	Wood, cognac	Other	932.4 ± 37.6	761.7 ± 74.5	701.5 ± 39.2
	Methyl salicylate	Esters	Mint, spices	Spices	3.2 ± 1.0	<1	<1
	4-Vinylguaicaol	Benzenoids	Cloves, curry	Spices	55.3 ± 4.4	23.3 ± 10.5	2.1 ± 0.6
	Phenylacetaldehyde	Benzenoids	Orange flowers, honey	Floral	28.5 ± 4.9	15.7 ± 8.3	16.4 ± 1.8
	4-Carbethoxy butyrolactone	Lactones			56.9 ± 3.3	49.2 ± 8.4	43.3 ± 1.5
**T2-n characterizing**	Isoamyl acetate	Esters	Banana	Fruity	312.3 ± 76.5	765.6 ± 169.3	269.5 ± 11.5
	β-Phenylethyl acetate	Esters	Rose, honey	Floral	31.23 ± 8.6	114.1 ± 31.8	47.2 ± 6.8
	Ethyl butanoate	Esters	Kiwifruit, pineapple	Fruity	29.9 ± 1.4	79.9 ± 30.2	39.5 ± 11.9
	Ethyl hexanoate	Esters	Apple	Fruity	98.4 ± 14.9	293.6 ± 79.9	170.1 ± 16.8
	^*^Ethyl 4-hydroxybutanoate	Esters	Fruity, honey	Fruity	2.6 ± 0.5	39.6 ± 1.3	15.3 ± 2.3
	Methyl vanillate	Esters	Green tea	Spices	2.8 ± 0.3	4.8 ± 0.7	1.8 ± 0.2
	trans-3-Hexen-1-ol	Alcohols	Apple, herbal	Fruity	11.3 ± 2.4	29.9 ± 5.1	6.1 ± 1.4
	Benzyl alcohol	Alcohols	Fruity, balsamic	Fruity	64.8 ± 24.7	735.4 ± 37.6	124.8 ± 24.5
	Furfuryl alcohol	Alcohols			2.5 ± 0.4	8.2 ± 0.3	5.5 ± 1.0
	Homovanillyl alcohol	Alcohols			64.6 ± 22.4	418.9 ± 39.8	1.6 ± 0.4
	Guaiacol	Benzenoids	Smoked	Spices	<1	1.6 ± 0.2	<1
	γ-butyrolactone	Lactones	Peach	Fruity	282.6 ± 20.6	979.1 ± 99.9	561.2 ± 69.9
	Decanoic acid	Carboxylic acids	Cheese	Cheesy	214.7 ± 1.6	563.9 ± 104.8	253.9 ± 35.4
	Homovanillic acid	Carboxylic acids			1.2 ± 0.4	8.6 ± 1.6	<1
**T2-i characterizing**	Ethyl phenylacetate	Esters	Rose, honey, tobacco	Floral	5.0 ± 0.5	3.1 ± 0.7	7.4 ± 0.8
	Ethyl 2-hydroxyvalerate	Esters	Banana	fruity	3.0 ± 0.8	1.7 ± 0.7	4.2 ± 0.5
	Diethyl succinate	Esters	Fruity	Fruity	56.4 ± 5.5	58.6 ± 9.5	135.3 ± 13.3
	Isoamyl lactate	Esters			5.5 ± 1.6	13.3 ± 2.4	20.8 ± 5.5
	Ethyl isoamylsuccinate	Esters			1.5 ± 0.5	<1	4.8 ± 0.4
	Diethyl maleate	Esters			29.4 ± 1.2	33.7 ± 3.7	66.5 ± 14.7
	Ethyl vanillate	Esters	Vanilla	Spices	3.9 ± 0.8	1.9 ± 0.6	10.1 ± 1.2
	2-Hexen-1-ol	Alcohols			<1	<1	2.5 ± 0.9
	1-Octen-3-ol	Alcohols	Mushrooms	Other	<1	<1	17.0 ± 1.7
	trans-Linalool oxide C	Terpenes	Floral	Floral	<1	<1	2.7 ± 0.2
	Ho-diendiol 1	Terpenes	Muscat, white moss	Floral	15.2 ± 6.2	23.3 ± 2.8	37.5 ± 1.4
	4-Terpineol	Terpenes	Lilac, earthy, underwood	Floral	<1	29.1 ± 13.6	432.5 ± 122.2
	p-Cresol	Benzenoids	Stable	Off-flavor	<1	1.7 ± 0.1	9.9 ± 2.1
	Vanillin	Benzenoids	Vanilla	Spices	2.2 ± 0.1	8.4 ± 3.4	70.5 ± 22.6
	Phenol	Benzenoids	Smoked	Spices	1.9 ± 0.1	8.30 ± 1.1	15.7 ± 1.1
	Benzaldehyde	Benzenoids	Almond	Spices	2.7 ± 0.5	22.4 ± 13.5	580.4 ± 87.1
	γ-nonalactone	Lactones	Coconut	Fruity	5.4 ± 0.6	7.5 ± 0.9	17.2 ± 1.6
	Sherry lactone 1	Lactones	Spice	Spices	114.1 ± 20.4	219.7 ± 12.7	933.2 ± 96.2
	Sherry lactone 2	Lactones	Spice	Spices	701.9 ± 89.6	407.0 ± 19.2	^*^1.0 ± ^*^0.3
	N-(3-Methylbutyl)-acetamide	Amides	Pungent (winegar)	Off-flavor	79.3 ± 4.5	158.7 ± 137.1	^*^19.9 ± ^*^6.3

*The corresponding pq(corr) values are reported in Supplementary File 3. Items marked with an asterisk have to be multiplied by 10^3^*.

When the two withering processes were compared, wine from the naturally withered berries was strongly characterized by the presence of ethyl-4-hydroxy butanoate, as well as benzyl alcohol, eugenol, guaiacol, homovanillic alcohol, homovanillic acid, trans-3-hexenol, β-damascenone, and methyl vanillate. In contrast, the botrytized wine was strongly characterized by the presence of N-(3-methylbutyl)acetamide, as well as sherry lactone 1, benzaldehyde, 1-octen-3-ol, trans-8-dihydroxylinalool, ethyl vanillate, ethyl isoamyl succinate, diethyl succinate, p-cresol, ho-diendiol, 4-terpineol, γ-nonalactone, and ethyl phenylacetate (Figure 5D and Supplementary File 3). When considering only the more abundant VOCs (more than 500 ppb) showing at least a two-fold difference in abundance between the two samples, the wine produced from naturally-withered berries was characterized by isovalerianic acid, isoamylacetate, decanoic acid, and homovanillic acid, whereas the botrytized wine was characterized by N-(3-methylbutyl)-acetamide, sherry lactones 1 and 2, benzaldehyde and 4-terpineol.

## Discussion

### The natural development of noble rot can be strongly induced in Garganega berries undergoing postharvest dehydration

The postharvest induction of noble rot could be used for the production of botrytized wines in regions with climates unsuitable for natural botrytization and also in those with suitable climates, to overcome the unpredictability of natural botrytization. However, a controlled widespread noble rot development on dehydrating grapes is not easy to achieve because the natural or forced ventilation of cases to accelerate dehydration makes the berries less susceptible to infection with *B. cinerea* (Barbanti et al., 2008). Fedrizzi et al. (2011) investigated natural botrytization in Corvina berries during withering, but in this case it was necessary to discard rotten berries developing gray mold and to manually separate the botrytized and non-botrytized fruit. Lorenzini et al. (2012) showed that noble rot can be induced under postharvest laboratory conditions by inoculating Garganega and Corvina berries with the fungus. The ability to achieve widespread noble rot development during natural withering has been reported anecdotally (Ferrarini et al., 2009; Vannini and Chilosi, 2013).

Here we demonstrated the ability to induce noble rot development in Garganega berries without the concomitant development of gray mold by implementing a special management strategy during postharvest withering, comprising an initial period of normal withering to allow partial berry dehydration (which prevents the development of gray mold by ensuring the adequate concentration of sugars) followed by a period of increased humidity achieved by covering the berries in the presence of water-filled trays. This simple procedure increased the humidity without affecting the temperature, slightly reduced the rate of berry dehydration, and encouraged the development of *B. cinerea* infection without the need for artificial inoculation because the fungus is commonly present in the vineyard and in cellars as an environmental contaminant. Noble rot induction was confirmed by berry characteristics and the enumeration of *B. cinerea* CFUs in selective medium. We characterized the changes in the metabolite profile of grapes and wines attributable to the proliferation of the fungus. However, the possibility that the modified air humidity could be the cause of part of the differences between T2-n and T2-i cannot be completely ruled out.

### *B. cinerea* growth and plant defence can be monitored by untargeted metabolomics

Untargeted metabolomics based on LC-MS revealed metabolites associated with the infection of berries by *B. cinerea*. Some of these metabolites were biochemical markers of the fungus, including structural components and products of fungal metabolism, while others were derived from the berries and represent the onset of plant defense mechanisms during withering.

The LC-MS data matrix revealed many imprints of fungal metabolism, including the presence of the N-acetylglucosamine donor uridine 5′-diphospho-N-acetylglucosamine, which is utilized by fungi including *B. cinerea* as a substrate for the enzyme chitin synthase (Causier et al., 1994). The declining levels of many grape metabolites in botrytized fruits suggests they were degraded by fungal metabolism, including sucrose, hydroxycinnamic acids (coutaric, caftaric, and fertaric acids), amino acids, lignans, and many flavonoids (including flavan-3-ols and flavonols). The loss of polyphenols has been reported in other white-berry cultivars infected with noble rot, including Chenin Blanc (Carbajal-Ida et al., 2016) and Chardonnay (Hong et al., 2012) although there was a specific increase in the abundance of flavan-3-ols in Chenin Blanc, in contrast to other polyphenols (Carbajal-Ida et al., 2016). However, cultivars such as Sémillon accumulated high levels of phenylpropanoids following the onset of noble rot (Blanco-Ulate et al., 2015). Therefore, the impact of *B. cinerea* on phenylpropanoid metabolism appears to be cultivar dependent.

The stilbenes are phytoalexins that are known to accumulate during botrytization (Landrault et al., 2002; Blanco-Ulate et al., 2015). The observed decline in the abundance of stilbene monomers (resveratrol and resveratrol glucoside) could reflect the consumption of these metabolites by the fungus, but the concomitant increase in the levels of stilbene dimers, trimers and tetramers suggests that botrytization causes the aggregation of stilbene monomers into oligomers. The accumulation of the oxylipin 13-KODE could also represent a plant defense response because this metabolite is induced as a defense molecule in soybean (*Glycine max*) in response to fungi such as *Aspergillus niger, A. oryzae, Rhizopus oligosporus*, and *A. niger* wry (Feng et al., 2007). To the best of our knowledge, this is the first report describing the induction of 13-KODE in grapevine berries in response to noble rot. However, octadecadienoic acids, the precursors of KODE oxylipins, have been proposed as potential positive metabolic markers of gray mold (Agudelo-Romero et al., 2015). The botrytized fruits also accumulated large amounts of pantothenic acid, D-glucose-6-phosphate and two unannotated lipids.

### The postharvest induction of noble rot influenced the accumulation of wine aroma compounds

The proliferation of *B. cinerea* induced remarkable changes in the accumulation of VOCs, affecting several aroma compounds that may contribute to the sensory characters of white wines. From a quantitative perspective, N-(3-methylbutyl)acetamide was the strongest marker of botrytized wine in agreement with previous studies of botrytization in Recioto di Soave (Azzolini et al., 2013; Tosi et al., 2013), Amarone (Fedrizzi et al., 2011), and Fiano (Genovese et al., 2007) wines. From a qualitative perspective, several VOCs detected at lower concentrations but with a potentially higher impact on aroma (Francis and Newton, 2005) were also influenced by noble rot. The sherry lactone isomers and γ-nonalactone were detected at higher concentrations in botrytized wines, in agreement with previous reports (Genovese et al., 2007; Sarrazin et al., 2007; Azzolini et al., 2013; Tosi et al., 2013). Although lactones do not contribute directly to the aroma of botrytized wines, they are involved in perceptive interaction phenomena resulting in an enhanced sensory contribution, e.g., synergy between γ-nonalactone and eugenol can enhance the overripe orange aroma notes typical of noble rot wines (Stamatopoulos et al., 2014). Eugenol is mostly derived from contact between the wine and oak wood, so the storage in oak barrels of Garganega wines from berries infected with noble rot could enhance these overripe orange aromas. Terpenes such as citronellol, ho-diendiol, hydroxylated linalool derivatives and 4-terpineol became more abundant during dehydration in the berries with noble rot, especially in the case of 4-terpineol. Likewise, the norisoprenoid 3-oxo-α-ionol (which gives rise to the tobacco aroma compound megastigmatrienone) accumulated to higher levels during withering. Terpenes and norisoprenoids are two important groups of aroma compounds that contribute the floral, fruity and tobacco-like attributes of wines. They accumulate in the berries as free molecules and as glycosylated precursors, which can be revealed by the action of yeast during fermentation or by acid hydrolysis during wine aging (Ugliano et al., 2006). Dehydration can favor their accumulation, and the presence of *B. cinerea* can facilitate their release from precursors by means of the pool of enzymes released into the must (Donèche, 1993).

Several volatile benzenoids, generally characterized by sweet/spicy aroma notes, were shown to increase in response to noble rot and/or simple dehydration, including benzaldehyde, vanillin, cresols, guaiacols and eugenol. Although benzaldehyde is often associated with the development of *B. cinerea* (Genovese et al., 2007; Fedrizzi et al., 2011), the behavior of volatile benzenoids has not been investigated in detail. However, Genovese et al. (2007) also observed the accumulation of eugenol and vinyl guaiacol in white wines prepared from berries infected with noble rot. The mushroom-like aroma compound 1-octen-3-ol is found at significantly higher concentrations in wines produced from botrytized berries. *B. cinerea* and other pathogens such as *Uncinula necator* (powdery mildew) produce 1-octen-3-ol, which at high concentrations can reduce the quality of berries and introduce mushroom off-odors in the finished wine (Darriet et al., 2002).

Interestingly, several aroma compounds arising from yeast metabolism, in particular the powerful fruit-smelling esters isoamyl acetate, ethyl butanoate, ethyl hexanoate and ethyl octanoate, were present at lower concentrations in the botrytized wines compared to wines from either the fresh or dehydrated berries. When comparing fresh and dehydrated berries, it was clear that dehydration without noble rot infection favored the accumulation of these metabolites during fermentation, probably due to the higher concentrations of nitrogen available to the yeast (Ugliano et al., 2006; Vilanova et al., 2007). The lower concentrations of esters in the botrytized wines could therefore reflect the depletion of nitrogen or the release of esterases by *B. cinerea*.

In conclusion, the analysis of the volatile fraction of wines and evaluation of the potential odor contribution of different volatiles indicated that wines from dehydrated berries were generally characterized by higher content of fresh fruit-smelling compounds (esters), whereas noble rot induced the accumulation of several spicy aroma compounds such as lactones, combined with compounds with floral attributes such as 4-terpineol and the mushroom smelling compound 1-octen-3-ol.

## Author contributions

GT: designed the experiments; SN and FB: performed the LC-MS-based untargeted metabolomics; SN and RD: performed the GC-MS based metabolomics; MC: performed the lipidomic experiment; ST and ES: did the bacterial counts and analyses; AP and AL: projected and executed the sampling and the agronomical analyses; SN and FG: analyzed the metabolomics data; FG: wrote the manuscript; SN, AL, MU, and GT: contributed to the draft writing; MU, AP, and ST: critically revised the manuscript; all authors read and approved the final manuscript.

### Conflict of interest statement

The authors declare that the research was conducted in the absence of any commercial or financial relationships that could be construed as a potential conflict of interest.
