# Supplementary figures and images for: The Induction of Noble Rot (Botrytis cinerea) Infection during Postharvest Withering Changes the Metabolome of Grapevine Berries (Vitis vinifera L., cv. Garganega)
Source: Front Plant Sci. 2017 Jun 21;8:1002. doi: 10.3389/fpls.2017.01002 (PMC5478704; doi:10.3389/fpls.2017.01002)

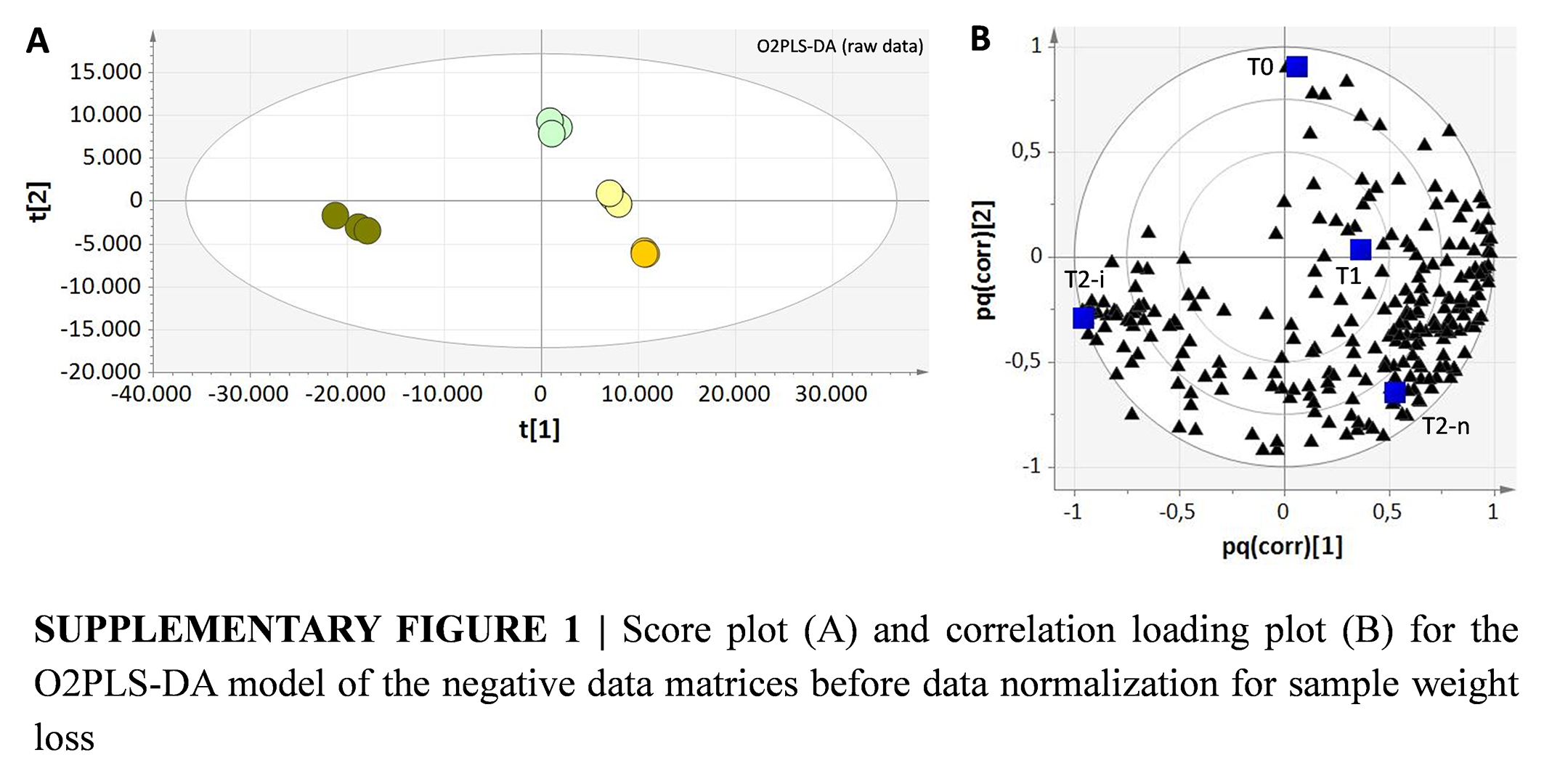

Supplement: Supplementary file 4 [file Image1.TIF]

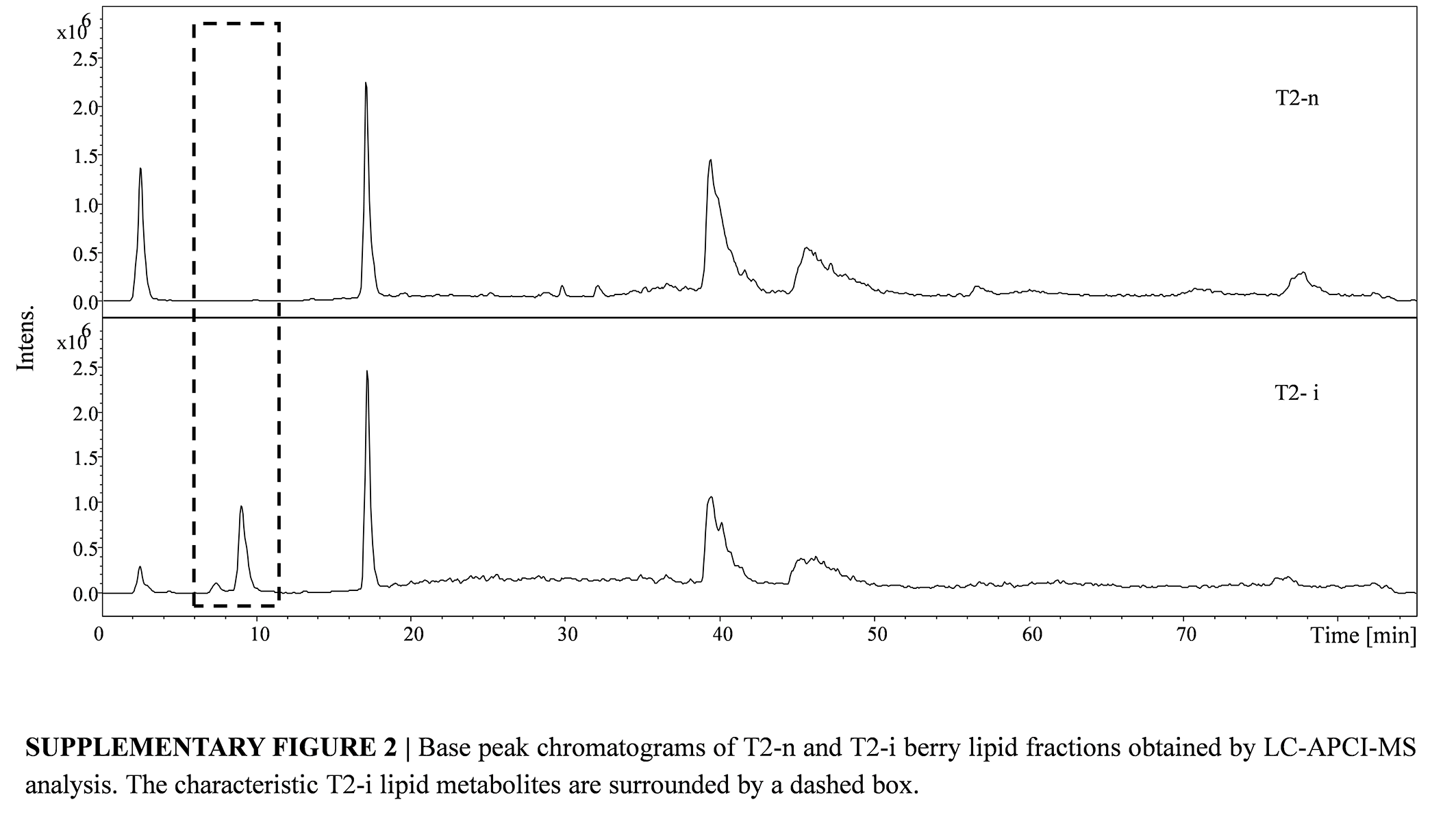

Supplement: Supplementary file 5 [file Image2.TIF]
